# SWATH-Based Comprehensive Determination of the Localization of Apical and Basolateral Membrane Proteins Using Mouse Liver as a Model Tissue

**DOI:** 10.3390/biomedicines10020383

**Published:** 2022-02-05

**Authors:** Satoshi Hirano, Ryohei Goto, Yasuo Uchida

**Affiliations:** Division of Membrane Transport and Drug Targeting, Graduate School of Pharmaceutical Sciences, Tohoku University, 6-3 Aoba, Aramaki, Aoba-ku, Sendai 980-8578, Japan; fire.ssvv@gmail.com (S.H.); ryohei.goto.0707@gmail.com (R.G.)

**Keywords:** basolateral membrane, apical membrane, membrane localization, comprehensive quantitative proteomics, SWATH

## Abstract

The purpose of this study was to develop a method to comprehensively determine the localization of apical and basolateral membrane proteins, using a combination of apical/basolateral membrane separation and accurate SWATH (**S**equential **W**indow **A**cquisition of all **TH**eoretical fragment ion spectra) proteomics. The SWATH analysis of basolateral and apical plasma membrane fractions in mouse liver quantified the protein expression of 1373 proteins. The basolateral/apical ratios of the protein expression levels were compared with the reported immunohistochemical localization for 41 model proteins (23 basolateral, 11 apical and 7 both membrane-localized proteins). Three groups were perfectly distinguished. Border lines to distinguish the apical-, both- and basolateral localizations were determined to be 0.766 and 1.42 based on probability density. The method that was established was then applied to the comprehensive determination of the proteins in mouse liver. The findings indicated that 154 and 125 proteins were localized in the apical and basolateral membranes, respectively. The levels of receptors, CD antigens and integrins, enzymes and Ras-related molecules were much higher in apical membranes than in basolateral membranes. In contrast, the levels of adhesion molecules, scaffold proteins and transporters in basolateral membranes were much higher than in apical membranes.

## 1. Introduction

Tissues harbor two types of cell membranes, namely, apical and basolateral membranes. The membrane facing basal lamina and neighboring cells is referred to as the “basolateral membrane”. The membrane on the opposite side of the basolateral membrane in polarized cells is referred to as the “apical membrane”. Elucidating which proteins are expressed on which membranes is important in many areas of biology. For example, transporters typically transport substances in a fixed direction, and the direction of transport can be 180 degrees different depending on which membrane they are expressed on. Therefore, to understand the physiological and pharmacological roles of an organ or a cell, it is essential to understand the localization of the transporter. In terms of drug delivery, liposomes and other drug products that are administered via the bloodstream are taken up from the cell membrane on the blood side into the cells of each organ. For active targeting, membrane proteins that are localized on the blood side of the cell membrane need to be targeted. In contrast, to avoid side effects, it is desirable to target molecules that are not localized on the blood side of the cell membrane. In order to achieve this, it is important to have available a comprehensive list of membrane proteins that are localized on apical and basolateral cell membranes in each organ. This task is best suited to comprehensive profiling approaches such as proteomics, rather than laborious immunohistochemical analysis.

Since the liver is the organ where drug delivery system (DDS) products such as liposomes tend to accumulate, it is important that the products are not easily taken up by liver cells in order to avoid toxicity. The ASGPR receptor is localized on the blood side of the cell membrane of liver parenchyma cells. The uptake of DDSs that target this receptor on liver cells are dramatically increased [1]. In contrast, the surface binding of folic acid has been reported to reduce the uptake of DDS products into the liver [2]. We have only limited knowledge regarding the localization of folate receptors and transporters in the liver, but our current knowledge suggests that they may be localized to apical membranes (not the blood side). Thus, a comprehensive determination of the localization of many proteins on basolateral and apical membranes would accelerate the development of drugs that have minimal or no liver side effects or are targeted to the liver.

The SWATH method is one of the more recent comprehensive quantitative proteomics methods that have been developed, and its excellent quantitative accuracy as compared with previous comprehensive proteomics is a significant advantage [3]. Multiple specific peptides derived from a single protein can be quantified, and the change in the level of expression of a target protein can be accurately quantified based on the average of these peptides. Membrane proteins contain hydrophobic regions, such as transmembrane sites, which cause their incomplete solubilization and resistance to trypsin digestion. However, we have previously reported on an improvement in the accuracy of the SWATH method by completely solubilizing such proteins with guanidine hydrochloride, thus improving the efficiency of the tryptic digestion of membrane proteins [4], and by applying in silico peptide selection criteria [5], such as excluding transmembrane sites and sequences that are known to be poorly digested by trypsin from the numerous peptides that are measured [6,7]. Furthermore, it has been known for a long time that apical and basolateral membranes can be fractionated from tissue samples based on the difference in the density of the cell membrane [8]. We hypothesized that, if the SWATH analysis could be applied to fractions of apical and basolateral membranes fractionated from each organ, it would be possible to comprehensively and accurately determine the localization of many membrane proteins in a variety of organs.

The purpose of this study was to demonstrate that by employing a combination of plasma membrane fractionation and the SWATH method, it would be possible to comprehensively and accurately determine the apical/basolateral localization of membrane proteins, using mouse liver as a model organ. In this study, a list of proteins localized on the apical and basolateral membranes in mouse liver was generated by this comprehensive localization analysis.

## 2. Materials and Methods

### 2.1. Animals

Male ddY mice were purchased from Charles River (Yokohama, Japan). The mice were maintained on a 12 h light/dark cycle in a temperature-controlled environment with free access to food and water. ddY mice were used at 10 weeks of age. The animal experiments were conducted based on ARRIVE guidelines, and the protocol was approved by the Institutional Animal Care and Use Committee at Tohoku University.

### 2.2. Separation of Basolateral (Sinusoidal) and Apical (Canalicular) Plasma Membranes of Mouse Liver by Density-Gradient Ultracentrifugation

The separation of basolateral and apical plasma membranes was performed as previously described with minor modifications [4]. The mouse liver was excised after PBS perfusion from the heart under anesthesia and were minced well with scissors on ice and homogenized by 10 up-and-down rotated strokes (1000 rpm) of a Potter-Elvehjem glass homogenizer in 40 mL of hypotonic buffer (10 mM Tris–HCl (pH 7.4), 10 mM NaCl, 1.5 mM MgCl_2_, 1 mM phenylmethylsulfonyl fluoride (PMSF) and a protease inhibitor cocktail (1% (*v*/*v*), Sigma Chemical Co., St. Louis, MO, USA)) per g wet tissue on ice. After incubation for 30 min on ice, 20 up-and-down strokes with rotation (1000 rpm, 4 °C) were applied. The resulting homogenate was subjected to nitrogen cavitation at 800 psi for 15 min at 4 °C twice. The resulting homogenate was centrifuged at 8000× *g* for 10 min at 4 °C and the resulting supernatant was centrifuged at 100,000× *g* for 60 min at 4 °C. The pellet was suspended in suspension buffer (10 mM Tris-HCl, 250 mM sucrose, pH 7.4), layered on top of a 38% (*w/v*) sucrose density gradient solution and centrifuged at 100,000× *g* for 40 min at 4 °C. The turbid layer was recovered, diluted in suspension buffer and centrifuged at 100,000× *g* for 40 min at 4 °C. The resultant pellet (used as the plasma membrane fraction) was suspended in 4 mL of suspension buffer and homogenized using a 10 mL glass homogenizer (50 up-and-down strokes with rotation at 4 °C). The homogenate was layered on top of a 31%/34%/38% (*w/v*) sucrose density gradient solution, and centrifuged at 195,700× *g* for 3 h at 4 °C. The two turbid layers at the interfaces (the first layer, top/31%, apical plasma membrane fraction; the third layer, 34%/38%, basolateral plasma membrane fraction) were recovered, and each was diluted in suspension buffer and centrifuged at 100,000× *g* for 40 min at 4 °C. The resultant pellets were suspended in suspension buffer to give the individual membrane fractions. The Lowry method with the DC protein assay reagent (Bio-Rad Laboratories, Hercules, CA, USA) was used to measure protein concentrations. The membrane fractions were stored at −80 °C.

### 2.3. Sample Preparation for SWATH-Based Quantitative Proteomics

Protein digestion of basolateral and apical membrane fractions prepared from four mouse livers was performed using 50 μg of protein per tube as described previously [9]. The tryptic digests were cleaned up with a self-packed SDB-XD 200 µL tip (3M, Maplewood, MN, USA), as previously described [10].

### 2.4. LC-MS/MS Measurement for SWATH-Based Quantitative Proteomics

The cleaned peptide samples (1 μg peptide) were injected into an NanoLC 425 system (Eksigent Technologies, Dublin, CA, USA) coupled with an electrospray-ionization Triple TOF 5600 mass spectrometer (SCIEX, Framingham, MA, USA), which was set up for a single direct injection and analyzed by SWATH-MS acquisition, as previously described [3] with minor modifications. The peptides were directly loaded onto a self-packed 20 cm long C18 analytical column, prepared by packing ProntoSIL 200-3-C18 AQ beads (Catalogue number 0001H184PS030, 3 μm, 120Å, BISCHOFF Chromatography, Germany) in a PicoFrit tip (ID 75 μm, Catalogue number PF360-75-10-N5, New Objective, Littleton, MA, USA). After sample loading, the peptides were separated and eluted with a linear gradient; 98% A: 2% B to 65% A: 35% B (0–120 min), increase to 0% A: 100% B (120–121 min), maintained at 0% A: 100% B (121–125 min), reduced to 98% A: 2% B (125–126 min) and then maintained at 98% A: 2% B (126–155 min). The composition of Mobile phase A was 0.1% formic acid in water, and that for mobile phase B was 0.1% formic acid in acetonitrile. The flow rate was 300 nL/min. The eluted peptides were positively ionized and measured in the SWATH mode. The measurement parameters were as follows: SWATH window, 64 variable windows from 400 m/z to 1200 m/z; product ion scan range, 50–2000 m/z; declustering potential, 100; rolling collision energy value, 0.0625 × [m/z of each SWATH window]−3.5; collision energy spread, 15; accumulation time, 0.05 s for each SWATH window.

### 2.5. Data Analysis for SWATH-Based Quantitative Proteomics

Spectral alignment and data extraction from the SWATH chromatogram (uploaded to the Peptide Atlas website with Identifier PASS01726) were performed with the SWATH Processing Micro App in Peakview (SCIEX) using in-house spectral libraries (uploaded to the Peptide Atlas website with Identifier PASS01726), as previously described [7]. The parameters for peak data extraction by Peakview were as follows: number of peptides per protein, 999; number of transitions per peptide, 6; peptide confidence threshold, 99%; false discovery rate (FDR) threshold, 1.0%; XIC extraction window, ±4.0 min; XIC width (ppm), 50. According to a previously described procedure [6], unreliable peaks and peptides were removed based on the criteria of data selection and amino acid sequence-based peptide selection, and the peak areas at the peptide level were calculated as an average of those in the transition level after normalizing the differences in signal intensity between the different transitions. The peptide selection criteria are described in Table S1. The details were reported in our previous study [6]. The peak areas of individual proteins were calculated as an average of those at the peptide level, and were used to calculate the basolateral/apical (B/A) ratio. The mean and SEM of four mice were calculated. Subcellular location information for all the proteins quantified was obtained from the uniport database.

## 3. Results

### 3.1. Validation of the SWATH-Based Comprehensive Determinations of Basolateral and Apical Plasma Membrane Localizations

**Figure 1:**
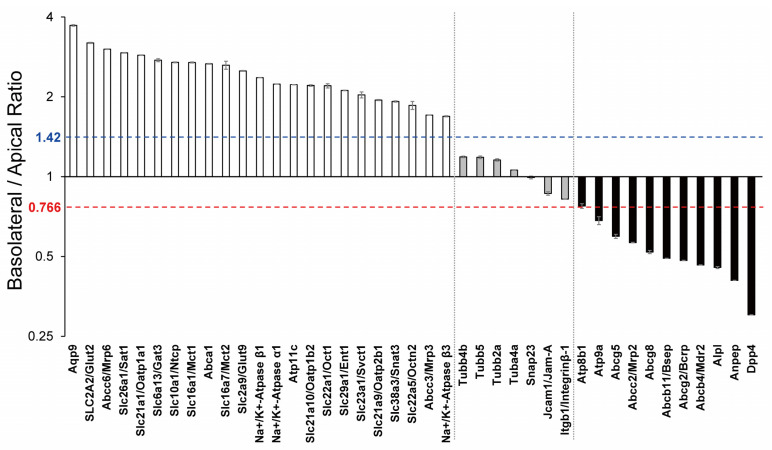
Comparison of the reported immunohistochemical localization and the basolateral/apical ratios that were comprehensively determined by SWATH analysis in this study. The basolateral/apical ratios of proteins whose membrane localizations were previously clarified by immunohistochemical analysis in liver are shown. The columns represent the mean ± SEM (*n* = 4). White column, proteins reported to be localized at the basolateral plasma membrane in liver; grey column, proteins reported to be localized at both basolateral and apical plasma membranes in the liver; black column, proteins reported to be localized at the apical plasma membrane in liver. Literature information concerning the membrane localization of individual molecules, Aqp9 [11], SLC2A2/Glut2 [12], Abcc6/Mrp6 [13], Slc26a1/Sat1 [14], Slc21a1/Oatp1a1 [13], Slc6a13/Gat3 [15], Slc10a1/Ntcp [13], Slc16a1/Mct1 [16], Abca1 [13], Slc16a7/Mct2 [17], Slc2a9/Glut9 [18], Na^+^/K^+^-Atpase β1 [19], Na^+^/K^+^-Atpase α1 [19], Atp11c [20], Slc21a10/Oatp1b2 [13], Slc22a1/Oct1 [13], Slc29a1/Ent1 [13], Slc23a1/Svct1 [21], Slc21a9/Oatp2b1 [13], Slc38a3/Snat3 [22], Slc22a5/Octn2 [23], Abcc3/Mrp3 [13], Na^+^/K^+^-Atpase β3 [19], Tubulins [24], Snap23 [25], Jcam1/Jam-A [26], Itgb1/Integrinβ-1 [27], Atp8b1 [13], Atp9a [28], Abcg5 [13], Abcc2/Mrp2 [13], Abcg8 [13], Abcb11/Bsep [13], Abcg2/Bcrp [13], Abcb4/Mdr2 [13], Alpl [29], Anpep [29] and Dpp4 [29]. Significant differences were observed between the basolateral-localized (white) and both-localized (grey) groups (*p* = 9.82 × 10^−7^, Kolmogorov–Smirnov test), between both-localized (grey) and apical-localized (black) groups (*p* = 1.00 × 10^−4^, Kolmogorov–Smirnov test), and between basolateral-localized (white) and apical-localized (black) groups (*p* = 6.99 × 10^−9^, Kolmogorov–Smirnov test). Furthermore, the mean and variance of 7 both-localized proteins were 1.04 and 0.0227, respectively (using 7 mean values but not using 7 × 4 (28 values)). The variances (*n* = 4) in B/A ratios were also calculated for each protein t is localized at basolateral (23 proteins) and apical (11 proteins) membranes. Using these values, a Student’s *t*-test followed by a Bonferroni correction showed that each B/A ratio for 23 basolateral- and 11 apical-localized proteins is significantly different from the distribution of B/A ratios of 7 both-localized proteins (Bonferroni-adjusted *p* value < 0.05). Furthermore, on the basis of probability density (Figure S1), the border lines of three groups were drawn at 0.766 and 1.42.

SWATH analysis of basolateral and apical plasma membrane fractions in mouse liver showed that 1373 proteins were quantified (Table S2). Basolateral/Apical (B/A) ratios and subcellular locations based on the uniprot database for 1373 proteins are shown in Table S2. The B/A ratios for proteins reported to be localized at apical or basolateral plasma membranes in the liver are presented (Figure 1). Significant differences in the B/A ratios were observed between 23 basolateral-localized (white column) and 7 both-localized (grey column) proteins (*p* = 9.82 × 10^−7^, Kolmogorov–Smirnov test), between 7 both-localized (grey column) and 11 apical-localized (black column) proteins (*p* = 1.00 × 10^−4^, Kolmogorov–Smirnov test), and between 23 basolateral-localized (white column) and 11 apical-localized (black column) proteins (*p* = 6.99 × 10^−9^, Kolmogorov–Smirnov test). Furthermore, the mean and variance of 7 both-localized proteins in Figure 1 were 1.04 and 0.0227, respectively (using 7 mean values but not using 7 × 4 (28 values)). The variances (*n* = 4) in B/A ratios were also calculated for each protein that is localized at basolateral (23 proteins) and apical (11 proteins) membranes. To show whether the B/A ratios of 23 basolateral- and 11 apical-localized proteins are statistically significantly different from distribution of both-localized protein group, the B/A ratios of individual basolateral and apical proteins were compared with the distribution of B/A ratios of 7 both-localized proteins by using a Student’s *t*-test followed by a Bonferroni correction. It showed that each B/A ratio for 23 basolateral- and 11 apical-localized proteins is significantly different from the distribution of B/A ratios of 7 both-localized proteins (Bonferroni-adjusted *p* value < 0.05) (Figure 1). Based on the values of B/A ratio of 11 apical-, 7 both- and 23 basolateral-localized proteins, the mean and standard deviation of B/A ratio of each of the three groups were calculated and then the probability density was estimated by modeling the B/A ratios as log-normally distributed (Figure S1; the *p*-value for the Shapiro–Wilk test for normality on the logged B/A ratios were 0.959, 0.228 and 0.869 for apical-, both- and basolateral-localized groups, respectively). The intersection of the normal distributions of apical- and both-localized protein groups was 0.766. The intersection of the normal distributions for the basolateral- and both-localized protein groups was 1.42. These values would be used as border lines of B/A ratios to distinguish apical, both and basolateral localizations.

### 3.2. SWATH-Based Comprehensive Determinations of Basolateral and Apical Plasma Membrane Localizations in Mouse Liver

Based on the results of the validation described above, we can assume that molecules with a B/A ratio of less than 0.766 are localized to apical plasma membranes and molecules with a B/A ratio of more than 1.42 are localized to basolateral plasma membranes. To avoid the inclusion of molecules that are localized to organelle membranes in the cell, we focused on molecules that have been shown to be localized to cell membranes in the uniprot database (molecules including “cell membrane” as an uniprot subcellular location keyword). There are molecules, however, whose cell membrane localization is not registered in the uniprot database. Among the proteins not including “cell membrane” but including “membrane”, the proteins including the keywords organelle membrane other than “cell membrane” (e.g., mitochondrial membrane) were deleted, and the remaining proteins were selected as proteins that are potentially located in the plasma membrane. In other words, from the total 1373 proteins in Table S2, we selected molecules that that are localized to cell membranes and molecules that could potentially be localized to cell membranes, and the molecules with a B/A ratio of less than 0.766 (apical) or greater than 1.42 (basolateral) are listed in Table 1 and Table 2, respectively. The B/A ratios of individual proteins listed in Table 1 and Table 2 were compared with the distribution of B/A ratios of 7 both-localized proteins in Figure 1 by using a Student’s *t*-test followed by a Bonferroni correction. It showed that each B/A ratio for all the proteins is significantly different from the distribution of B/A ratios of 7 both-localized proteins (Bonferroni-adjusted *p* value < 0.05) (Table 1, Table 2, Tables S3 and S4).

It was estimated that 154 proteins are localized to the apical membrane of the mouse liver (Table 1). These included 17 receptors, 3 GPCR-related molecules, 11 CD antigens, 4 integrins, 2 adhesion molecules, 16 transporters, 2 channels, 6 pumps, 2 scaffold proteins, 1 proteoglycan, 42 enzymes, 3 Rho molecules, 9 Ras-related molecules, 32 other molecules and 4 uncharacterized molecules. Folr2 and slc46a1, which are involved in folate transport, were also included. A total of 125 proteins were estimated to be localized in the basolateral membrane of mouse liver cells (Table 2), including 11 receptors, 1 GPCR-related molecule, 2 CD antigens, 7 adhesion molecules, 34 transporters, 2 channels, 4 pumps, 10 scaffold proteins, 11 enzymes, 1 Rho molecule, 41 other molecules and 1 uncharacterized molecule. Scarb1, Asgr1 and Asgr2 were also included. A comparison of the data in Table 1 and Table 2 shows that the number of membrane proteins, such as receptors, CD antigens and integrins, is much higher in apical membranes than in basolateral membranes. Similarly, the number of enzymes and Ras-related molecules was also much higher in the apical membrane. In contrast, there were considerably more adhesion molecules, scaffold proteins and transporters in basolateral membranes than in apical membranes.

## 4. Discussion

In this study, we report on the development of a method for determining the apical and basolateral localization of membrane proteins in a comprehensive manner that does not involve the use of antibodies. In this study, we evaluated the accuracy of the SWATH method using 23, 11 and 7 proteins, which have been reported to be localized to the apical, the basolateral and both plasma membranes of hepatocytes, respectively, as model molecules. The results showed that apical and basolateral membrane proteins could be clearly distinguished (Figure 1). Thus, the method proved to be highly accurate and comprehensive in determining the apical and basolateral localization of membrane proteins.

The list of molecules shown in Table 1 and Table 2 will be useful in terms of drug delivery. The selective delivery of drugs to the liver is important in the treatment of liver diseases. The targeted delivery of oligonucleotides to liver hepatocytes using N-acetylgalactosamine (GalNAc) conjugates that bind to the asialoglycoprotein receptor (Asgr) has become a breakthrough approach in the field of therapeutic oligonucleotides [1]. Although it is known that Asgr is localized on basolateral membranes, in this study, we were able to separately quantify Asgr1 and Asgr2, and the results clearly shows that both molecules are localized on basolateral membranes (Table 2). Regarding Asgr, it has been reported that Asgr-mediated delivery can become saturated [30]. As shown in Table 2, not only Asgr1 and Asgr2, but also the Scarb1, Ptprf, Adra1b, Ptprg, Lsr, Lsr, Ptprd, Egfr, Insr and Erbb3 receptors are localized to the basolateral membrane. Not only receptors, but also CD antigens, integrins, transporters and other membrane proteins can be internalized by the binding of ligands such as antibodies. Therefore, it is hoped that this list will be of use in terms of drug delivery for liver diseases. Several types of membrane protein internalization mechanisms are known, including clathrin-mediated and caveolin-mediated types. In the basolateral membrane of the liver, many molecules that are involved in clathrin-mediated endocytosis, such as the AP-2 complex (Ap2b1, Ap2m1, Ap2a2, Ap2s1 and Ap2a1), Picalm, Dnm2 and Clint1 were localized. Therefore, among the molecules that are localized to basolateral membranes, those that internalize in a clathrin-dependent manner may be useful for the smooth efficient delivery of drugs to the liver. In contrast, the localization of caveolin-1 in apical membranes (Table 1) suggests that caveolin-mediated internalization may be more active in apical membranes.

In contrast, most drugs (which can be toxic) tend to accumulate in the liver. Therefore, it is important to establish a drug delivery system that does not transfer drugs to normal hepatocytes, but, rather, to diseased tissue. In normal hepatocytes, DDS products in blood cannot access the membrane proteins of the apical membrane because of the presence of tight junctions. Therefore, membrane proteins that are localized to the apical membrane in normal hepatocytes (Table 1) may be promising receptors for DDS systems that avoid hepatotoxicity. Folr2 and slc46a1, which prefer folate as a ligand or substrate, have been shown to become localized to the apical membrane (Table 1). The binding of folic acid to the surface of liposomes has been reported to decrease the number of liposomes that are transferred to the liver and, therefore, to increase the amount transferred to cancerous tissues [2]. This suggests that when considering active targeting to tissues other than the liver, targeting membrane proteins that are present on the surface of the relevant tissue but are not localized to the basolateral membrane of the liver may increase the amount transferred to the target tissue and decrease the amount transferred to the liver.

Interestingly, many of the low molecular weight G-protein Rab molecules that are involved in epithelial polarity transport were found to be localized to the apical membrane (Table 1; Rab1A, Rab5a, Rab5b, Rab5c, Rab8a, Rab9a, Rab18 and Rab35). In general, Rab molecules are involved in intracellular vesicular trafficking to the apical membranes, where they support the localization of membrane proteins to the apical membrane. Although the Rab subtypes in hepatocytes have not been fully elucidated, the findings reported in this study indicate that the above Rab subtypes are localized to the apical membrane. In contrast, no Rab molecules were detected in the basolateral membrane fraction (Table 2). It will be interesting to determine whether these Rab subtypes are involved in vesicular trafficking to the apical membrane in hepatocytes. However, further research would be needed in the future to validate the estimated localizations listed in Table 1 and Table 2 by immunohistochemistry.

Researching DDSs that deliver cargoes into the central nervous systems (CNS), such as the brain and spinal cord, is an important issue. Given the fact that the blood–brain barrier and the blood–arachnoid barrier are large surface area barriers, it would be desirable for DDS products to pass through these barriers. Methods for separating the blood- and CNS-side plasma membranes of barrier cells have been reported. Briefly, after the isolation of blood vessels or leptomeninges from the brain or spinal cord, the blood- and CNS-side plasma membranes can be fractionated by density gradient centrifugation using different concentrations of ficoll or sucrose [4,31]. It is also important to list molecules that are not localized to the blood-side plasma membranes of the intestinal and renal epithelial cells to avoid adverse effects due to transfer to these organs, which have a high blood flow. Methods for fractionating the blood- and luminal-side plasma membranes of each epithelial cell have been established [32,33,34,35]. By combining the cell membrane fractionation methods for each of these tissues with the SWATH method, an exhaustive list of molecules that are localized or are not localized to the blood-side plasma membrane can be generated with a high degree of accuracy and comprehensiveness.

## 5. Conclusions

**Figure 2:**
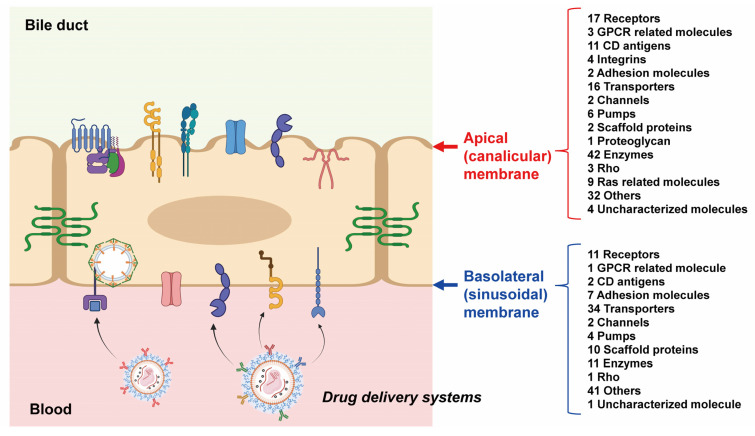
Schematic illustration of the apical and basolateral localization of proteins in mouse hepatocyte determined in this study. The apical and basolateral localization of proteins are illustrated based on the data of Table 1 and Table 2, respectively. In physiologically normal conditions, the drug delivery systems administered into the systemic circulation usually access the basolateral surface of hepatocytes. Therefore, the membrane proteins that are localized at the basolateral membrane potentially support the cellular uptake drugs from drug delivery systems.

By combining the conventional apical/basolateral membrane separation method with high precision SWATH proteomics, it was possible to comprehensively determine the localization of apical and basolateral membrane proteins (Figure 1 and Figure 2). In terms of drug delivery, it is hoped that this method will be found to be applicable to other organs and human tissues in the future, so that a list of proteins that are localized on membranes of all organs can be established.

## Figures and Tables

**Table 1:** Functional classification of membrane proteins that are abundantly expressed in apical membrane fractions.

Protein Name	Uniprot Accession Number	B/A Ratio (Mean ± SEM)	Plasma Membrane Llocalized? (Based on Uniprot)	Protein Name	Uniprot Accession Number	B/A Ratio (Mean ± SEM)	Plasma Membrane Localized? (Based on Uniprot)	Protein Name	Uniprot Accession Number	B/A Ratio (Mean ± SEM)	Plasma Membrane Localized? (Based on Uniprot)
***Receptors***				***Channels***				***Rho***			
Folr2	Q05685	0.367 ± 0.010	Yes	Tmem63a	Q91YT8	0.490 ± 0.013	Yes	Cdc42	P60766	0.501 ± 0.004	Yes
Rpsa	P14206	0.407 ± 0.001	Yes	Aqp1	Q02013	0.694 ± 0.004	Yes	Rhog	P84096	0.526 ± 0.005	Yes
Stra6l	Q9DBN1	0.483 ± 0.002	Yes					Rhob	P62746	0.643 ± 0.011	Yes
Fcgr2	P08101	0.483 ± 0.004	Yes	***Pumps***							
Ptprc	P06800	0.496 ± 0.003	Yes	Atp6v1e1	P50518	0.383 ± 0.010	Yes	***Ras related molecules***			
Calcrl	Q9R1W5	0.503 ± 0.006	Yes	Atp6v1b2	P62814	0.513 ± 0.004	Yes	Rab1A	P62821	0.460 ± 0.003	Potentially
Fcer1g	P20491	0.518 ± 0.009	Yes	Tcirg1	Q9JHF5	0.574 ± 0.001	Potentially	Rab9a	Q9R0M6	0.463 ± 0.005	Yes
Npr1	P18293	0.518 ± 0.010	Potentially	Atp6v1d	P57746	0.592 ± 0.008	Potentially	Rab35	Q6PHN9	0.479 ± 0.002	Yes
P2rx4	Q9JJX6	0.552 ± 0.002	Potentially	Atp2b4	Q6Q477	0.611 ± 0.006	Potentially	Rab5a	Q9CQD1	0.620 ± 0.008	Yes
Mrc1	Q61830	0.554 ± 0.000	Yes	Atp6v0a2	P15920	0.690 ± 0.008	Yes	Rras	P10833	0.648 ± 0.004	Yes
Rack1	P68040	0.588 ± 0.002	Yes					Rab5c	P35278	0.653 ± 0.013	Yes
Il6st	Q00560	0.607 ± 0.007	Yes	***Scaffold proteins***				Rab18	P35293	0.660 ± 0.008	Yes
Stab1	Q8R4Y4	0.628 ± 0.001	Potentially	Cav1	P49817	0.584 ± 0.006	Yes	Rab5b	P61021	0.708 ± 0.012	Yes
Stab2	Q8R4U0	0.634 ± 0.001	Yes	Msn	P26041	0.732 ± 0.003	Yes	Rab8a	P55258	0.733 ± 0.010	Yes
Fcgrt	Q61559	0.655 ± 0.003	Yes								
Pigr	O70570	0.715 ± 0.001	Yes	***Proteoglycan***				***Others***			
Prlr	Q08501	0.746 ± 0.006	Potentially	Sdc4	O35988	0.340 ± 0.005	Potentially	Susd2	Q9DBX3	0.437 ± 0.005	Yes
								Mreg	Q6NVG5	0.437 ± 0.007	Yes
***GPCR related molecules***				***Enzymes***				Meak7	Q8K0P3	0.458 ± 0.004	Potentially
Gnai2	P08752	0.449 ± 0.002	Yes	Dpep1	P31428	0.273 ± 0.006	Yes	Lamtor1	Q9CQ22	0.470 ± 0.002	Yes
Gpr155	A2AWR3	0.527 ± 0.003	Potentially	Bst1	Q64277	0.290 ± 0.002	Yes	Ehd3	Q9QXY6	0.475 ± 0.004	Yes
Gpr182	P43142	0.715 ± 0.010	Yes	Dpp4	P28843	0.301 ± 0.000	Yes	Lmbrd1	Q8K0B2	0.523 ± 0.008	Yes
				Cd38	P56528	0.334 ± 0.001	Potentially	Gpc4	P51655	0.523 ± 0.012	Yes
***CD antigens***				Entpd8	Q8K0L2	0.386 ± 0.004	Yes	Epb41l2	O70318	0.568 ± 0.003	Yes
Lamp1	P11438	0.356 ± 0.001	Yes	Entpd1	P55772	0.400 ± 0.011	Potentially	Dysf	Q9ESD7	0.569 ± 0.001	Yes
Eng	Q63961	0.378 ± 0.002	Yes	Anpep	P97449	0.406 ± 0.001	Yes	Myof	Q69ZN7	0.584 ± 0.006	Yes
Cd44	P15379	0.405 ± 0.017	Yes	Enpp3	Q6DYE8	0.411 ± 0.009	Yes	Sidt2	Q8CIF6	0.586 ± 0.004	Yes
Lamp2	P17047	0.410 ± 0.001	Yes	Nt5e	Q61503	0.414 ± 0.002	Yes	Raet1d	Q9JI58	0.587 ± 0.014	Yes
Pecam1	Q08481	0.433 ± 0.005	Yes	Cemip2	Q5FWI3	0.438 ± 0.002	Yes	Clec4g	Q8BNX1	0.590 ± 0.002	Yes
Cd1d1	P11609	0.511 ± 0.005	Yes	Got2	P05202	0.444 ± 0.002	Yes	Irgm1	Q60766	0.599 ± 0.002	Yes
Bst2	Q8R2Q8	0.563 ± 0.002	Yes	Abhd17b	Q7M759	0.450 ± 0.020	Yes	Stx8	O88983	0.604 ± 0.003	Potentially
Cd36	Q08857	0.626 ± 0.006	Yes	Alpl	P09242	0.454 ± 0.004	Yes	Fam234b	Q8BYI8	0.640 ± 0.018	Potentially
Cd47	Q61735	0.686 ± 0.003	Yes	Nos3	P70313	0.475 ± 0.007	Yes	Atraid	Q6PGD0	0.657 ± 0.034	Yes
Cd59a	O55186	0.709 ± 0.013	Yes	Enpp4	Q8BTJ4	0.490 ± 0.005	Yes	Pttg1ip	Q8R143	0.657 ± 0.009	Yes
Cd68	P31996	0.760 ± 0.025	Yes	P4hb	P09103	0.510 ± 0.002	Yes	Gdi2	Q61598	0.661 ± 0.010	Potentially
				Plpp1	Q61469	0.514 ± 0.013	Yes	Hfe	P70387	0.678 ± 0.014	Yes
***Integrins***				Pip4p1	Q3TWL2	0.519 ± 0.004	Yes	Napa	Q9DB05	0.697 ± 0.003	Yes
Itga9	B8JK39	0.399 ± 0.014	Potentially	Pdia6	Q922R8	0.533 ± 0.001	Yes	Clec2d	Q91V08	0.704 ± 0.008	Yes
Itga1	Q3V3R4	0.622 ± 0.002	Potentially	Ece1	Q4PZA2	0.536 ± 0.002	Yes	Tmed1	Q3V009	0.717 ± 0.033	Yes
Itgal	P24063	0.648 ± 0.025	Yes	Adam23	Q9R1V7	0.552 ± 0.039	Yes	Mal2	Q8BI08	0.724 ± 0.002	Yes
Itgav	P43406	0.707 ± 0.003	Yes	Naalad2	Q9CZR2	0.557 ± 0.008	Yes	Kct2	Q8K201	0.730 ± 0.011	Potentially
				Enpep	P16406	0.561 ± 0.001	Yes	Hsp90aa1	P07901	0.746 ± 0.008	Yes
***Adhesion molecules***				Kars1	Q99MN1	0.575 ± 0.013	Yes	Itfg1	Q99KW9	0.750 ± 0.019	Potentially
Esam	Q925F2	0.615 ± 0.030	Yes	Mgll	O35678	0.590 ± 0.005	Potentially	Hpcal1	P62748	0.750 ± 0.017	Potentially
Icam2	P35330	0.701 ± 0.010	Potentially	Ggt6	Q6PDE7	0.610 ± 0.011	Potentially	Lrrc57	Q9D1G5	0.750 ± 0.018	Potentially
				Ctsb	P10605	0.629 ± 0.003	Yes	Tmem123	Q91Z22	0.756 ± 0.008	Potentially
***Transporters***				Akr1a1	Q9JII6	0.632 ± 0.007	Yes	Plin2	P43883	0.760 ± 0.005	Potentially
Slc46a3	Q9DC26	0.390 ± 0.004	Potentially	FRRS1	Q8K385	0.640 ± 0.008	Potentially	Rp2	Q9EPK2	0.765 ± 0.004	Yes
Slc39a4	Q78IQ7	0.414 ± 0.015	Yes	Cpd	O89001	0.649 ± 0.011	Yes				
Slc44a2	Q8BY89	0.449 ± 0.009	Potentially	B4galt1	P15535	0.650 ± 0.005	Yes	***Uncharacterized molecules***			
Abcb4	P21440	0.464 ± 0.001	Yes	Hpd	P49429	0.668 ± 0.001	Potentially	Tmem59	Q9QY73	0.291 ± 0.009	Yes
Abcg2	Q7TMS5	0.483 ± 0.001	Yes	C1galt1	Q9JJ06	0.675 ± 0.009	Potentially	Tmem176a	Q9DCS1	0.363 ± 0.009	Potentially
Abcb11	Q9QY30	0.492 ± 0.000	Yes	Adam10	O35598	0.683 ± 0.003	Yes	Tm9sf4	Q8BH24	0.578 ± 0.002	Potentially
Abcg8	Q9DBM0	0.520 ± 0.007	Yes	Eno1	P17182	0.684 ± 0.003	Yes	Tm7sf3	Q9CRG1	0.662 ± 0.012	Yes
Slc2a8	Q9JIF3	0.524 ± 0.017	Yes	Lnpep	Q8C129	0.690 ± 0.005	Yes				
Slc46a1	Q6PEM8	0.530 ± 0.005	Yes	Park7	Q99LX0	0.702 ± 0.006	Yes				
Abcb6	Q9DC29	0.532 ± 0.001	Yes	Pi4k2b	Q8CBQ5	0.723 ± 0.023	Potentially				
Abcc2	Q8VI47	0.564 ± 0.001	Yes	Pik3r4	Q8VD65	0.740 ± 0.033	Potentially				
Slc38a7	Q8BWH0	0.591 ± 0.006	Potentially	Nedd4	P46935	0.741 ± 0.005	Yes				
Abcg5	Q99PE8	0.596 ± 0.011	Yes	Cnp	P16330	0.754 ± 0.012	Potentially				
Slc12a9	Q99MR3	0.635 ± 0.007	Yes	Tgm2	P21981	0.765 ± 0.019	Yes				
Slc10a5	Q5PT54	0.704 ± 0.038	Potentially								
Slc30a10	Q3UVU3	0.750 ± 0.027	Yes								

Among the total 1373 proteins that were quantified (Table S2), proteins whose basolateral/apical (B/A) ratios were less than 0.766 were selected. Furthermore, proteins including the term “cell membrane” in the uniport subcellular location information were selected. On the other hand, among proteins not including “cell membrane” but including “membrane”, the proteins that included the keywords of organelle membranes other than cell membrane (e.g., mitochondrial membrane) were deleted, and the remaining proteins were selected as proteins that could be potentially located on the plasma membrane. The list of “cell membrane” proteins above was combined with that of proteins that could be potentially located on the plasma membrane. B/A ratio represents the mean ± SEM (*n* = 4). Furthermore, the mean and variance of 7 both-localized proteins in Figure 1 were 1.04 and 0.0227, respectively. The variances of the B/A ratios were also calculated for each protein in this table. Using these values, a Student’s *t*-test followed by a Bonferroni correction showed that each B/A ratio for all the proteins listed in this table is significantly different from the distribution of B/A ratios of 7 both-localized proteins (Bonferroni-adjusted *p* value < 0.05). The *p* values are listed in Table S3.

**Table 2:** Functional classification of membrane proteins that are abundantly expressed in basolateral membrane fractions.

Protein Name	Uniprot Accession Number	B/A Ratio (Mean ± SEM)	Plasma Membrane Localized? (Based on Uniprot)	Protein Name	Uniprot Accession Number	B/A Ratio (Mean ± SEM)	Plasma Membrane Localized? (Based on Uniprot)	Protein Name	Uniprot Accession Number	B/A Ratio (Mean ± SEM)	Plasma Membrane Localized? (Based on Uniprot)
Scarb1	Q61009	2.78 ± 0.02	Yes	Slc4a1	P04919	5.73 ± 0.06	Yes	Arhgef12	Q8R4H2	1.43 ± 0.05	Potentially
Ptprf	A2A8L5	1.98 ± 0.01	Potentially	Pdzk1	Q9JIL4	5.28 ± 0.01	Yes				
Adra1b	P97717	1.95 ± 0.03	Yes	Slc9a3r1	P70441	4.74 ± 0.03	Yes	***Others***			
Ptprg	Q05909	1.91 ± 0.07	Potentially	Slc2a2	P14246	3.19 ± 0.02	Yes	Actn1	Q7TPR4	5.68 ± 0.14	Yes
Lsr	Q99KG5	1.90 ± 0.01	Yes	Abca8a	Q8K442	3.09 ± 0.02	Yes	Utrn	E9Q6R7	5.31 ± 0.06	Yes
Ptprd	Q64487	1.64 ± 0.08	Potentially	Abcc6	Q9R1S7	3.02 ± 0.01	Yes	Stard10	Q9JMD3	4.83 ± 0.39	Potentially
Egfr	Q01279	1.62 ± 0.00	Yes	Slc26a1	P58735	2.93 ± 0.01	Yes	Tspan4	Q9DCK3	4.72 ± 0.14	Potentially
Insr	P15208	1.52 ± 0.01	Yes	Slc1a2	P43006	2.92 ± 0.04	Yes	Pacsin3	Q99JB8	4.32 ± 0.04	Yes
Erbb3	Q61526	1.45 ± 0.04	Potentially	Slco1a1	Q9QXZ6	2.87 ± 0.01	Yes	Cask	O70589	4.27 ± 0.03	Yes
Asgr1	P34927	1.43 ± 0.00	Potentially	Slc6a6	O35316	2.75 ± 0.03	Yes	Lima1	Q9ERG0	4.19 ± 0.03	Yes
Asgr2	P24721	1.43 ± 0.00	Potentially	Slc6a13	P31649	2.75 ± 0.04	Yes	Sntb1	Q99L88	3.81 ± 0.04	Yes
				Slc6a11	P31650	2.75 ± 0.03	Potentially	Scrib	Q80U72	3.65 ± 0.07	Yes
***GPCR related molecule***				Slc10a1	O08705	2.70 ± 0.01	Potentially	C2cd2	E9Q3C1	3.57 ± 0.08	Potentially
Gna12	P27600	1.66 ± 0.05	Yes	Slc16a1	P53986	2.69 ± 0.01	Yes	Dlg1	Q811D0	3.37 ± 0.04	Yes
				Abca1	P41233	2.66 ± 0.01	Yes	Dmd	P11531	3.07 ± 0.03	Yes
***CD antigens***				Slc16a7	O70451	2.63 ± 0.09	Yes	Farp1	F8VPU2	3.00 ± 0.03	Yes
Bsg	P18572	2.50 ± 0.01	Yes	Slc2a9	Q3T9X0	2.50 ± 0.01	Yes	Ttyh2	Q3TH73	2.76 ± 0.02	Yes
Cd82	P40237	2.37 ± 0.01	Yes	Slc4a4	O88343	2.42 ± 0.03	Yes	Fam126b	Q8C729	2.70 ± 0.05	Yes
				Abca8b	Q8K440	2.32 ± 0.01	Yes	Epb41l5	Q8BGS1	2.61 ± 0.06	Yes
***Adhesion molecules***				Atp11c	Q9QZW0	2.22 ± 0.00	Yes	Tspan9	Q8BJU2	2.59 ± 0.09	Potentially
Ctnnd1	P30999	2.21 ± 0.00	Yes	Slco1b2	Q9JJL3	2.21 ± 0.01	Yes	Twf1	Q91YR1	2.58 ± 0.04	Potentially
Ctnnb1	Q02248	2.20 ± 0.01	Yes	Slc22a1	O08966	2.20 ± 0.04	Yes	Ttc7a	Q8BGB2	2.55 ± 0.04	Yes
Ctnna1	P26231	2.01 ± 0.01	Yes	Slc29a1	Q9JIM1	2.11 ± 0.01	Yes	Coro1c	Q9WUM4	2.44 ± 0.02	Yes
Cdh2	P15116	1.93 ± 0.02	Yes	Slc30a1	Q60738	2.04 ± 0.02	Yes	Sptan1	P16546	2.41 ± 0.00	Yes
Cldnd1	Q9CQX5	1.75 ± 0.06	Potentially	Slc23a1	Q9Z2J0	2.03 ± 0.05	Yes	Pals2	Q9JLB0	2.36 ± 0.01	Potentially
Cadm1	Q8R5M8	1.74 ± 0.05	Yes	Slc39a14	Q75N73	2.00 ± 0.02	Yes	Serinc5	Q8BHJ6	2.35 ± 0.11	Yes
Cldn3	Q9Z0G9	1.64 ± 0.02	Yes	Slc12a7	Q9WVL3	1.95 ± 0.02	Yes	Nckap1	P28660	2.29 ± 0.04	Yes
				Slco2b1	Q8BXB6	1.94 ± 0.01	Yes	Eps15l1	Q60902	2.17 ± 0.05	Yes
***Scaffold proteins***				Slc38a3	Q9DCP2	1.92 ± 0.01	Yes	Wasf2	Q8BH43	2.13 ± 0.02	Yes
Rdx	P26043	2.72 ± 0.02	Yes	Slc22a5	Q9Z0E8	1.86 ± 0.06	Yes	Phb	P67778	2.11 ± 0.01	Yes
Ap2b1	Q9DBG3	2.05 ± 0.01	Yes	Slc22a23	Q3UHH2	1.82 ± 0.05	Potentially	Tmem30a	Q8VEK0	2.06 ± 0.01	Yes
Ezr	P26040	1.98 ± 0.04	Yes	Slc44a1	Q6X893	1.82 ± 0.02	Yes	Fam234a	Q8C0Z1	1.86 ± 0.01	Potentially
Picalm	Q7M6Y3	1.94 ± 0.02	Yes	Abcc3	B2RX12	1.71 ± 0.00	Yes	Numb	Q9QZS3	1.82 ± 0.01	Yes
Dnm2	P39054	1.74 ± 0.02	Potentially	Slc2a1	P17809	1.59 ± 0.05	Yes	Vapa	Q9WV55	1.77 ± 0.03	Yes
Ap2m1	P84091	1.68 ± 0.01	Yes					Stx4	P70452	1.76 ± 0.01	Yes
Ap2a2	P17427	1.66 ± 0.00	Yes	***Enzymes***				Efr3a	Q8BG67	1.74 ± 0.01	Yes
Ap2s1	P62743	1.65 ± 0.02	Yes	Cdc42bpb	Q7TT50	3.54 ± 0.01	Yes	Sema4g	Q9WUH7	1.72 ± 0.07	Yes
Clint1	Q99KN9	1.62 ± 0.01	Potentially	Zdhhc5	Q8VDZ4	2.59 ± 0.05	Yes	Eppk1	Q8R0W0	1.71 ± 0.03	Yes
Ap2a1	P17426	1.60 ± 0.01	Yes	Tgm1	Q9JLF6	2.43 ± 0.01	Potentially	Vcl	Q64727	1.68 ± 0.01	Yes
				Adcy9	P51830	2.16 ± 0.11	Yes	Plxnb2	B2RXS4	1.62 ± 0.00	Yes
***Channels***				Pi4ka	E9Q3L2	2.07 ± 0.01	Yes	Lrrn4	P59383	1.61 ± 0.09	Potentially
Aqp9	Q9JJJ3	3.72 ± 0.02	Yes	Atp5f1a	Q03265	1.86 ± 0.00	Yes	Stxbp3	Q60770	1.61 ± 0.01	Yes
Clic4	Q9QYB1	2.94 ± 0.03	Yes	Steap4	Q923B6	1.76 ± 0.01	Yes	Ndrg1	Q62433	1.50 ± 0.02	Yes
				Ilk	O55222	1.75 ± 0.03	Yes	Eps15	P42567	1.43 ± 0.02	Yes
***Pumps***				Enpp1	P06802	1.74 ± 0.01	Yes				
Fxyd1	Q9Z239	2.45 ± 0.02	Yes	Pik3c2a	Q61194	1.59 ± 0.07	Yes	***Uncharacterized molecules***			
Atp1b1	P14094	2.36 ± 0.00	Yes	Adam17	Q9Z0F8	1.55 ± 0.04	Yes	Tmem150a	Q91WN2	3.01 ± 0.10	Yes
Atp1a1	Q8VDN2	2.23 ± 0.00	Yes								
Atp1b3	P97370	1.69 ± 0.01	Yes								

Among the total 1373 proteins that were quantified (Table S2), the proteins whose basolateral/apical (B/A) ratios were more than 1.42 were selected. In addition, the proteins including “cell membrane” in uniport subcellular location information were selected. On the other hand, among proteins not including “cell membrane”, but including “membrane”, proteins including the keywords of organelle membrane other than cell membrane (e.g., mitochondrial membrane) were deleted, and the remaining proteins were selected as proteins that could be potentially located on the plasma membrane. The list of “cell membrane” proteins above was combined with that of proteins potentially located in plasma membrane. B/A ratio represents the mean ± SEM (*n* = 4). Furthermore, the mean and variance of 7 both-localized proteins in Figure 1 were 1.04 and 0.0227, respectively. The variances in B/A ratios were also calculated for each protein in this table. Using these values, a Student’s *t*-test followed by a Bonferroni correction showed that each B/A ratio for all the proteins listed in this table is significantly different from the distribution of B/A ratios of 7 both-localized proteins (Bonferroni-adjusted *p* value < 0.05). The *p* values are listed in Table S4.

## Data Availability

Raw MS data are available on the Peptide Atlas webpage with Identifier PASS01726.

## Supplementary Materials

The following are available online at https://www.mdpi.com/article/10.3390/biomedicines10020383/s1. Figure S1: Probability distribution based on 11 apical-, 7 both- and 23 basolateral-localized proteins. Table S1: In silico peptide selection criteria. Table S2: Ratios of the protein expression levels of all the proteins quantified by SWATH analysis between basolateral and apical membrane fractions in mouse liver. Table S3: The *p* values of the proteins listed in Table 1. Table S4: The *p* values of the proteins listed in Table 2.
