# Supplementary material for: SWATH-Based Comprehensive Determination of the Localization of Apical and Basolateral Membrane Proteins Using Mouse Liver as a Model Tissue
Source: Biomedicines. 2022 Feb 5;10(2):383. doi: 10.3390/biomedicines10020383 (PMC8962430; doi:10.3390/biomedicines10020383)
Supplement: Supplementary file 1 [file biomedicines-10-00383-s001.zip › biomedicines-1518897_SM-for xml.pdf]

Table S1. In silico peptide selection criteria.

	**In silico Peptide Selection Criteria**
1.	NOT including the transmembrane domain of the protein
2.	NOT including K or R except for the C terminal of the peptide
3.	NOT including KK, RR, RK, KR, KP or RP at the digestion site
4.	NOT including any modification except for C carbamidomethylation
5.	NOT including C residue without C carbamidomethylation
6.	NOT including M residue
7.	NOT including N or Q residue at the N terminal of the peptide
8.	NOT including NA or NG continuous sequence
9.	Peptide sequence specific to target protein among all proteins

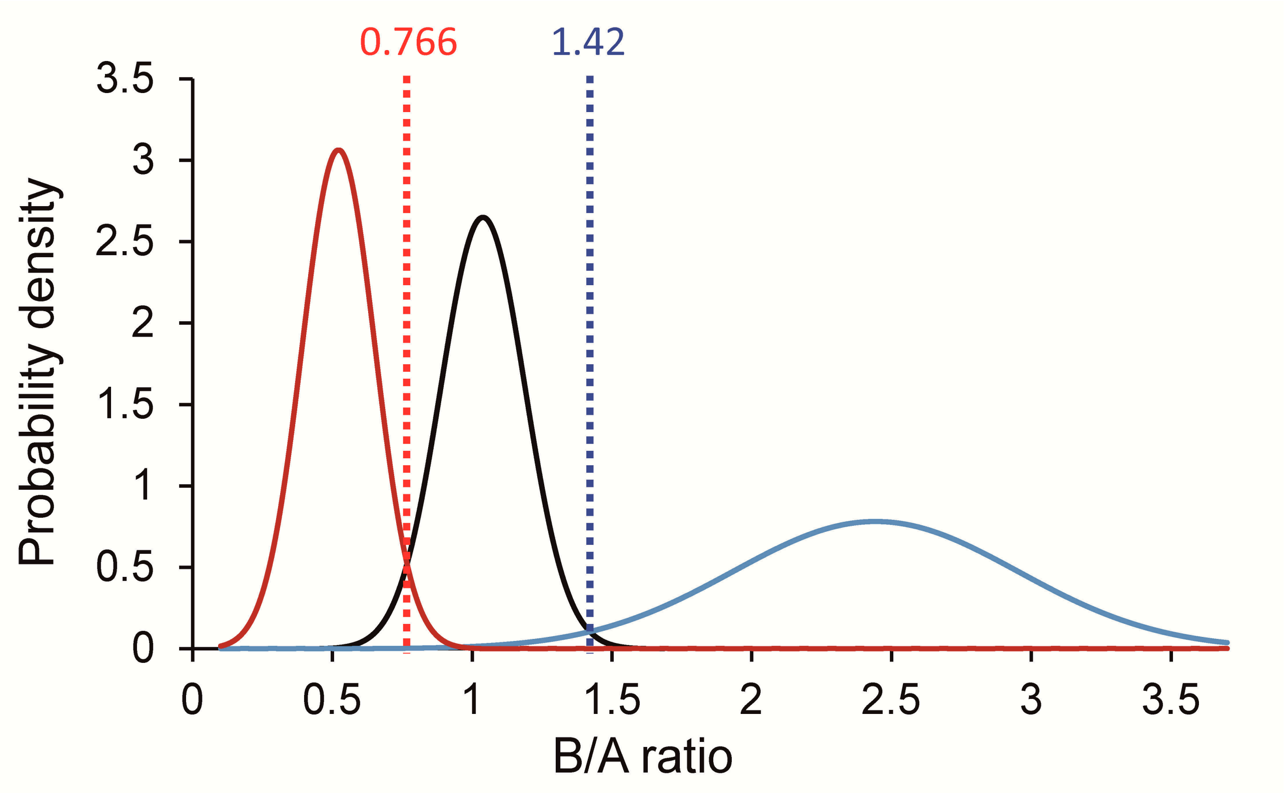

Figure S1. Probability distribution based on 11 apical-, 7 both- and 23 basolateral-localized proteins. Based on the values of B/A ratio of 11 apical-, 7 both- and 23 basolateral-localized proteins, the mean and standard deviation of B/A ratio of each of the three groups were calculated and then the probability density was estimated by modelling the B/A ratios as log-normally distributed. The p-value for the shapiro-wilk test for normality on the logged B/A ratios were 0.959, 0.228, and 0.869 for apical-, both- and basolateral-localized groups, respectively. The intersection of the normal distributions of apical- (red) and both- (black) localized protein groups was 0.766. The intersection of the normal distributions for the basolateral- (blue) and both- (black) localized protein groups was 1.42.
